# Aqua­[bis­(2-ethyl-5-methyl-1*H*-imidazol-4-yl-κ*N* ^3^)methane]­oxalatocopper(II) dihydrate

**DOI:** 10.1107/S1600536811001012

**Published:** 2011-01-15

**Authors:** Yang-Hui Luo, Xiao-Min Qian, Ge Gao, Jin-Feng Li, Shu-Lin Mao

**Affiliations:** a Ordered Matter Science Research Center, College of Chemistry and Chemical Engineering, Southeast University, Nanjing 210096, People’s Republic of China

## Abstract

In the title compound, [Cu(C_2_O_4_)(C_13_H_20_N_4_)(H_2_O)]·2H_2_O, the Cu^II^ atom exhibits a distorted square-pyramidal geometry with the two N atoms of the imidazole ligand and the two O atoms of the oxalate ligand forming the basal plane, while the O atom of the coordinated water mol­ecule is in an apical position. The Cu^II^ atom is shifted 0.232 (2) Å out of the basal plane toward the water mol­ecule. The asymmetric unit is completed by two solvent water mol­ecules. These water mol­ecules participate in the formation of an intricate three-dimensionnal network of hydrogen bonds involving the coordinated water mol­ecule and the NH groups.

## Related literature

For the chemical properties of imidazole derivatives, see: Bouwman *et al.* (2000 ▶). For synthesis, see: Delgado *et al.* (2008 ▶). For related structures, see: Beznischenko *et al.* (2007 ▶); Pajunen (1981 ▶). 
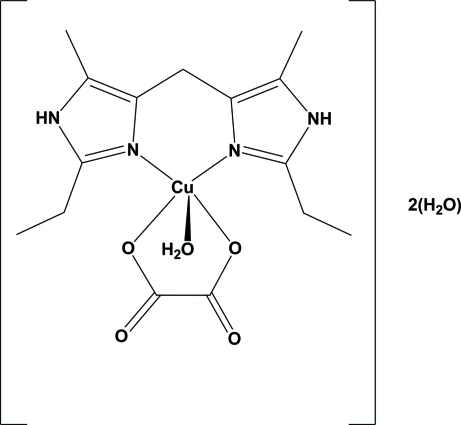


## Experimental

### 

#### Crystal data

[Cu(C_2_O_4_)(C_13_H_20_N_4_)(H_2_O)]·2H_2_O
*M* *_r_* = 437.94
Monoclinic, 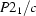

*a* = 12.1711 (13) Å
*b* = 23.167 (2) Å
*c* = 7.4400 (8) Å
β = 107.304 (1)°
*V* = 2002.9 (4) Å^3^
*Z* = 4
Mo *K*α radiation
μ = 1.13 mm^−1^
*T* = 298 K
0.35 × 0.18 × 0.12 mm

#### Data collection

Rigaku Mercury CCD area-detector diffractometer
Absorption correction: multi-scan (*CrystalClear*; Rigaku, 2005 ▶) *T*_min_ = 0.693, *T*_max_ = 0.876
10119 measured reflections
3528 independent reflections
1958 reflections with *I* > 2σ(*I*)
*R*_int_ = 0.071

#### Refinement

*R*[*F* ^2^ > 2σ(*F* ^2^)] = 0.045
*wR*(*F* ^2^) = 0.092
*S* = 0.81
3528 reflections
248 parameters
H-atom parameters constrained
Δρ_max_ = 0.34 e Å^−3^
Δρ_min_ = −0.31 e Å^−3^

### 

Data collection: *CrystalClear* (Rigaku, 2005 ▶); cell refinement: *CrystalClear*; data reduction: *CrystalClear*; program(s) used to solve structure: *SHELXS97* (Sheldrick, 2008 ▶); program(s) used to refine structure: *SHELXL97* (Sheldrick, 2008 ▶); molecular graphics: *ORTEPIII* (Burnett & Johnson, 1996 ▶), *ORTEP-3 for Windows* (Farrugia, 1997 ▶) and *PLATON* (Spek, 2009 ▶); software used to prepare material for publication: *SHELXL97*.

## Supplementary Material

Crystal structure: contains datablocks I, global. DOI: 10.1107/S1600536811001012/dn2649sup1.cif

Structure factors: contains datablocks I. DOI: 10.1107/S1600536811001012/dn2649Isup2.hkl

Additional supplementary materials:  crystallographic information; 3D view; checkCIF report

## Figures and Tables

**Table 1:** Hydrogen-bond geometry (Å, °)

*D*—H⋯*A*	*D*—H	H⋯*A*	*D*⋯*A*	*D*—H⋯*A*
O5—H51⋯O4^i^	0.85	2.58	3.177 (4)	128
O5—H52⋯O4^ii^	0.85	1.90	2.748 (4)	174
N2—H2⋯O2^iii^	0.86	2.09	2.943 (4)	171
N4—H4⋯O7	0.86	2.03	2.847 (4)	158
O6—H6*F*⋯O5^iv^	0.85	2.50	3.259 (4)	149
O6—H6*G*⋯O4^v^	0.85	2.30	3.057 (5)	148
O7—H7*C*⋯O3^vi^	0.85	2.03	2.882 (4)	178
O7—H7*D*⋯O6	0.85	1.97	2.818 (4)	177

Symmetry codes: (i) 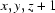; (ii) 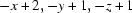; (iii) 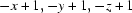; (iv) 
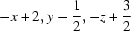
; (v) 
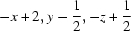
; (vi) 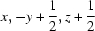.

**Fig. 1.**
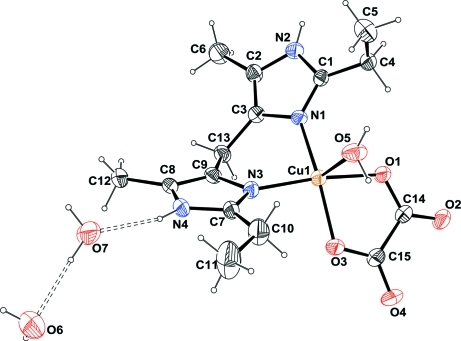
The asymmetric unit of (I) with the atom-labelling scheme. Displacement ellipsoids are drawn at the 30% probability level. H atoms are represented as small spheres of arbitrary radii. Hydrogen bonds are shown as dashed lines.

**Fig. 2.**
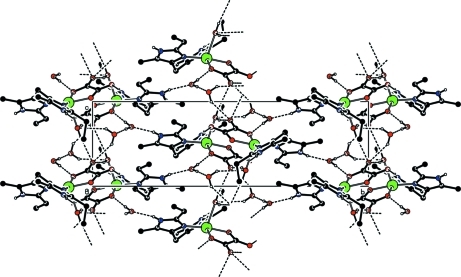
A packing view down the a axis showing the three dimensionnal network. Hydrogen bonds are shown as dashed lines. For the sake of clarity, H atoms not involved in hydrogen bonding have been omitted.

## supplementary crystallographic information

### Comment

Imidazole derivatives are versatile ligands towards transition metal ions both in man-made and natural systems. They are not only used for catalytic and biocatalysts but also for dioxygen transport and electron storage (Bouwman *et al.*, 2000). As part of our interest in imidazole derivatives, we report here the crystal structure of a new copper complex.

The structure around Cu^II^ is best decribed as distorted square pyramid environnement with the two N atoms of the imidazole ligand and the two O atoms of the oxalate forming the basal plane whereas the oxygen atom of the coordinated water molecule is in apical position. As expected, the copper atom is shifted ca 0.232 (2)Å out of the basal plane toward the water molecule. The asymmetric unit is completed by two solvate water molecules. The distances and angles within the square pyramid framework agree with related structures (Beznischenko *et al.*, 2007); Pajunen, 1981).

These water molecules participate to the formation of an intricated hydrogen bonds resulting in three dimensionnal network involving the coordinated water molecule and the NH groups (Table 1, Fig. 2).

### Experimental

Crystals of the title compound were synthesized by the reaction between copper(II) nitrate trihydrate, potassium oxalate and 4,4'-methanediylbis(2-ethyl-5-methyl-1*H*-imidazole) ligand. Copper salt and oxalate chemicals used (reagent grade) were commercially available, the 4,4'-methanediylbis(2-ethyl-5-methyl-1*H*-imidazole) ligand was synthesized as described below . 0.2 mmol(48.4 mg) solid copper(II) nitrate trihydrate was added to a 15 ml aqueous solution of 0.1 mmol(16.6 mg) potassium oxalate under continuous stirring. The suspension was heated at 40–50 °C during 1 h. Then this suspension was mixed with a 5 ml EtOH solution of 0.1 mmol(23.2mg)4,4'-methanediylbis(2-ethyl-5-methyl-1*H*-imidazole). Finally, the blue solution which results from the mixture was filtered off and allowed to evaporate at room temperature(Delgado, *et al.*,2008). Single crystals of the title compound as blue prisms were grown from the solution by slow evaporation at room temperature within a few days.

The ligand 4,4'-methanediylbis(2-ethyl-5-methyl-1*H*-imidazole) was synthesized as follows: 4.35 g (30 mmol) 2-ethyl-5-methylimidazole was added to a solution of 1.5 g (15 mmol)glycine (40% in H_2_O). This suspension was vigorously stirred, and 3.1 g (30 mmol) formaldehyde (37% in H_2_O) was added dropwise. The resulting turbid mixture was made alkaline with a concentrated sodium hydroxide solution until a pH of 12 was reached(Bouwman, *et al.*,2000). The reaction mixture was stirred for 8 days at room temperature in a closed vessel. During which time a white solid formed. The white solid was collected by filtration, washed with acetonitrile and diethyl ether, and air-dry at room temperature.

### Refinement

All H atoms attached to C atoms and N atom were fixed geometrically and treated as riding with C—H = 0.96 Å (methyl) or 0.97 Å (methylene) and N—H = 0.86 Å with U_iso_(H) = 1.2U_eq_(C or N) or U_iso_(H) = 1.5U_eq_(methyl) . H atoms of water molecules were located in difference Fourier maps and included in the subsequent refinement using restraints (O-H= 0.85 (1)Å and H···H= 1.40 (2)Å) with U_iso_(H) = 1.5U_eq_(O). In the last cycles of refinement, they were treated as riding on their parent O atoms.

### Crystal data

**Table:**

[Cu(C_2_O_4_)(C_13_H_20_N_4_)(H_2_O)]·2H_2_O	*F*(000) = 916
*M**_r_* = 437.94	*D*_x_ = 1.452 Mg m^−^^3^
Monoclinic, *P*2_1_/*c*	Mo *K*α radiation, λ = 0.71073 Å
Hall symbol: -P 2ybc	Cell parameters from 1383 reflections
*a* = 12.1711 (13) Å	θ = 3.0–26.0°
*b* = 23.167 (2) Å	µ = 1.13 mm^−^^1^
*c* = 7.4400 (8) Å	*T* = 298 K
β = 107.304 (1)°	Prism, blue
*V* = 2002.9 (4) Å^3^	0.35 × 0.18 × 0.12 mm
*Z* = 4	

### Data collection

**Table:**

Rigaku MODEL? CCD area-detector diffractometer	3528 independent reflections
Radiation source: fine-focus sealed tube	1958 reflections with *I* > 2σ(*I*)
graphite	*R*_int_ = 0.071
Detector resolution: 8.192 pixels mm^-1^	θ_max_ = 25.0°, θ_min_ = 2.5°
φ and ω scans	*h* = −14→14
Absorption correction: multi-scan (*CrystalClear*; Rigaku, 2005)	*k* = −27→21
*T*_min_ = 0.693, *T*_max_ = 0.876	*l* = −8→8
10119 measured reflections	

### Refinement

**Table:**

Refinement on *F*^2^	Primary atom site location: structure-invariant direct methods
Least-squares matrix: full	Secondary atom site location: difference Fourier map
*R*[*F*^2^ > 2σ(*F*^2^)] = 0.045	Hydrogen site location: inferred from neighbouring sites
*wR*(*F*^2^) = 0.092	H-atom parameters constrained
*S* = 0.81	*w* = 1/[σ^2^(*F*_o_^2^) + (0.0336*P*)^2^] where *P* = (*F*_o_^2^ + 2*F*_c_^2^)/3
3528 reflections	(Δ/σ)_max_ = 0.003
248 parameters	Δρ_max_ = 0.34 e Å^−^^3^
0 restraints	Δρ_min_ = −0.31 e Å^−^^3^

### Special details

**Table:**

Geometry. All esds (except the esd in the dihedral angle between two l.s. planes) are estimated using the full covariance matrix. The cell esds are taken into account individually in the estimation of esds in distances, angles and torsion angles; correlations between esds in cell parameters are only used when they are defined by crystal symmetry. An approximate (isotropic) treatment of cell esds is used for estimating esds involving l.s. planes.
Refinement. Refinement of *F*^2^ against ALL reflections. The weighted *R*-factor *wR* and goodness of fit *S* are based on *F*^2^, conventional *R*-factors *R* are based on *F*, with *F* set to zero for negative *F*^2^. The threshold expression of *F*^2^ > σ(*F*^2^) is used only for calculating *R*-factors(gt) *etc*. and is not relevant to the choice of reflections for refinement. *R*-factors based on *F*^2^ are statistically about twice as large as those based on *F*, and *R*- factors based on ALL data will be even larger.

### Fractional atomic coordinates and isotropic or equivalent isotropic displacement parameters (Å^2^)

**Table:**

	*x*	*y*	*z*	*U*_iso_*/*U*_eq_	
Cu1	0.76270 (4)	0.413233 (19)	0.51168 (7)	0.03980 (17)	
O1	0.7228 (2)	0.49415 (10)	0.4554 (4)	0.0521 (8)	
O2	0.7628 (2)	0.56946 (12)	0.2999 (4)	0.0607 (9)	
O3	0.8807 (2)	0.43059 (12)	0.3849 (4)	0.0489 (8)	
O4	0.9308 (3)	0.50045 (13)	0.2201 (5)	0.0744 (11)	
O5	0.8745 (2)	0.44573 (12)	0.8111 (4)	0.0598 (8)	
H51	0.8928	0.4356	0.9259	0.090*	
H52	0.9316	0.4637	0.7948	0.090*	
N1	0.6093 (2)	0.39498 (13)	0.5450 (4)	0.0350 (8)	
N2	0.4439 (3)	0.39461 (14)	0.6014 (5)	0.0454 (9)	
H2	0.3882	0.4056	0.6423	0.055*	
N3	0.8036 (3)	0.33050 (12)	0.5306 (5)	0.0392 (8)	
N4	0.8869 (3)	0.24674 (13)	0.5968 (5)	0.0435 (9)	
H4	0.9396	0.2212	0.6395	0.052*	
C1	0.5428 (3)	0.42338 (16)	0.6240 (5)	0.0355 (10)	
C2	0.4464 (3)	0.34484 (17)	0.5008 (6)	0.0455 (11)	
C3	0.5486 (3)	0.34484 (16)	0.4666 (6)	0.0365 (10)	
C4	0.5695 (3)	0.47801 (16)	0.7361 (6)	0.0441 (11)	
H4A	0.6252	0.5000	0.6946	0.053*	
H4B	0.4998	0.5009	0.7111	0.053*	
C5	0.6159 (4)	0.46803 (18)	0.9430 (7)	0.0665 (14)	
H5A	0.6874	0.4474	0.9698	0.100*	
H5B	0.5617	0.4458	0.9850	0.100*	
H5C	0.6284	0.5045	1.0073	0.100*	
C6	0.3461 (3)	0.30308 (19)	0.4455 (7)	0.0729 (16)	
H6A	0.3218	0.2942	0.5538	0.109*	
H6B	0.3696	0.2683	0.3973	0.109*	
H6C	0.2835	0.3203	0.3503	0.109*	
C7	0.9028 (3)	0.30417 (17)	0.6091 (6)	0.0403 (10)	
C8	0.7732 (4)	0.23503 (16)	0.5055 (6)	0.0417 (11)	
C9	0.7212 (3)	0.28746 (16)	0.4628 (6)	0.0385 (10)	
C10	1.0158 (3)	0.33196 (18)	0.7043 (7)	0.0669 (15)	
H10A	1.0293	0.3614	0.6207	0.080*	
H10B	1.0092	0.3514	0.8161	0.080*	
C11	1.1157 (4)	0.2954 (2)	0.7594 (10)	0.116 (3)	
H11A	1.1282	0.2785	0.6494	0.173*	
H11B	1.1038	0.2654	0.8408	0.173*	
H11C	1.1816	0.3180	0.8248	0.173*	
C12	0.7272 (3)	0.17517 (16)	0.4687 (6)	0.0559 (13)	
H12A	0.6454	0.1766	0.4111	0.084*	
H12B	0.7438	0.1544	0.5854	0.084*	
H12C	0.7626	0.1560	0.3860	0.084*	
C13	0.5996 (3)	0.30202 (16)	0.3626 (6)	0.0450 (11)	
H13A	0.5540	0.2669	0.3434	0.054*	
H13B	0.5952	0.3176	0.2398	0.054*	
C14	0.7802 (3)	0.51976 (19)	0.3595 (6)	0.0420 (11)	
C15	0.8731 (4)	0.48180 (19)	0.3153 (6)	0.0465 (11)	
O6	1.1845 (3)	0.07904 (14)	0.6127 (5)	0.0904 (11)	
H6F	1.1814	0.0493	0.6779	0.136*	
H6G	1.1552	0.0701	0.4975	0.136*	
O7	1.0089 (3)	0.14122 (14)	0.7024 (6)	0.1067 (15)	
H7C	0.9722	0.1194	0.7563	0.160*	
H7D	1.0608	0.1214	0.6762	0.160*	

### Atomic displacement parameters (Å^2^)

**Table:**

	*U*^11^	*U*^22^	*U*^33^	*U*^12^	*U*^13^	*U*^23^
Cu1	0.0386 (3)	0.0339 (3)	0.0511 (3)	0.0007 (2)	0.0197 (3)	0.0014 (3)
O1	0.0566 (19)	0.0352 (17)	0.078 (2)	0.0074 (13)	0.0411 (18)	0.0105 (15)
O2	0.066 (2)	0.041 (2)	0.084 (3)	0.0000 (15)	0.0355 (19)	0.0150 (17)
O3	0.0421 (17)	0.046 (2)	0.066 (2)	0.0043 (13)	0.0285 (16)	0.0026 (15)
O4	0.079 (2)	0.064 (2)	0.105 (3)	−0.0064 (17)	0.067 (2)	0.012 (2)
O5	0.0454 (18)	0.084 (2)	0.051 (2)	−0.0148 (15)	0.0156 (16)	−0.0019 (16)
N1	0.0313 (18)	0.037 (2)	0.039 (2)	0.0026 (14)	0.0142 (17)	0.0010 (15)
N2	0.033 (2)	0.047 (2)	0.060 (3)	0.0009 (16)	0.0204 (18)	0.0009 (18)
N3	0.036 (2)	0.033 (2)	0.050 (2)	0.0015 (16)	0.0155 (18)	0.0029 (16)
N4	0.044 (2)	0.035 (2)	0.055 (3)	0.0080 (16)	0.0182 (19)	0.0012 (17)
C1	0.035 (2)	0.033 (3)	0.038 (3)	0.0006 (19)	0.009 (2)	0.0045 (19)
C2	0.039 (3)	0.041 (3)	0.055 (3)	−0.002 (2)	0.012 (2)	−0.006 (2)
C3	0.029 (2)	0.040 (3)	0.037 (3)	−0.0010 (18)	0.006 (2)	−0.0020 (19)
C4	0.045 (3)	0.035 (3)	0.057 (3)	0.0029 (19)	0.022 (2)	0.000 (2)
C5	0.070 (3)	0.065 (3)	0.061 (4)	0.013 (3)	0.014 (3)	−0.006 (3)
C6	0.046 (3)	0.068 (4)	0.109 (5)	−0.016 (2)	0.032 (3)	−0.021 (3)
C7	0.040 (3)	0.031 (3)	0.052 (3)	0.003 (2)	0.017 (2)	0.000 (2)
C8	0.057 (3)	0.034 (2)	0.043 (3)	−0.002 (2)	0.028 (2)	−0.007 (2)
C9	0.040 (3)	0.040 (3)	0.038 (3)	−0.002 (2)	0.015 (2)	−0.008 (2)
C10	0.042 (3)	0.052 (3)	0.100 (5)	0.003 (2)	0.011 (3)	−0.001 (3)
C11	0.051 (4)	0.068 (4)	0.205 (8)	0.010 (3)	0.004 (4)	0.008 (4)
C12	0.065 (3)	0.038 (3)	0.073 (4)	−0.005 (2)	0.034 (3)	−0.011 (2)
C13	0.044 (3)	0.042 (3)	0.050 (3)	−0.003 (2)	0.016 (2)	−0.010 (2)
C14	0.040 (3)	0.042 (3)	0.042 (3)	−0.007 (2)	0.011 (2)	0.003 (2)
C15	0.043 (3)	0.046 (3)	0.053 (3)	−0.009 (2)	0.017 (2)	−0.001 (2)
O6	0.086 (3)	0.076 (3)	0.114 (3)	0.0166 (19)	0.038 (2)	−0.001 (2)
O7	0.119 (3)	0.070 (2)	0.167 (4)	0.045 (2)	0.098 (3)	0.062 (2)

### Geometric parameters (Å, °)

**Table:**

Cu1—O1	1.951 (2)	C4—H4B	0.9700
Cu1—N3	1.975 (3)	C5—H5A	0.9600
Cu1—O3	1.980 (3)	C5—H5B	0.9600
Cu1—N1	2.000 (3)	C5—H5C	0.9600
Cu1—O5	2.362 (3)	C6—H6A	0.9600
O1—C14	1.283 (4)	C6—H6B	0.9600
O2—C14	1.230 (4)	C6—H6C	0.9600
O3—C15	1.287 (4)	C7—C10	1.493 (5)
O4—C15	1.216 (4)	C8—C9	1.363 (5)
O5—H51	0.8489	C8—C12	1.490 (5)
O5—H52	0.8497	C9—C13	1.485 (5)
N1—C1	1.311 (4)	C10—C11	1.437 (5)
N1—C3	1.407 (4)	C10—H10A	0.9700
N2—C1	1.342 (4)	C10—H10B	0.9700
N2—C2	1.380 (5)	C11—H11A	0.9600
N2—H2	0.8599	C11—H11B	0.9600
N3—C7	1.323 (4)	C11—H11C	0.9600
N3—C9	1.398 (4)	C12—H12A	0.9600
N4—C7	1.344 (4)	C12—H12B	0.9600
N4—C8	1.375 (5)	C12—H12C	0.9600
N4—H4	0.8603	C13—H13A	0.9700
C1—C4	1.497 (5)	C13—H13B	0.9700
C2—C3	1.341 (5)	C14—C15	1.543 (5)
C2—C6	1.515 (5)	O6—H6F	0.8505
C3—C13	1.501 (5)	O6—H6G	0.8503
C4—C5	1.491 (6)	O7—H7C	0.8504
C4—H4A	0.9700	O7—H7D	0.8495
			
O1—Cu1—N3	171.72 (13)	C2—C6—H6A	109.5
O1—Cu1—O3	82.66 (11)	C2—C6—H6B	109.5
N3—Cu1—O3	91.60 (12)	H6A—C6—H6B	109.5
O1—Cu1—N1	92.73 (11)	C2—C6—H6C	109.5
N3—Cu1—N1	90.62 (12)	H6A—C6—H6C	109.5
O3—Cu1—N1	159.73 (12)	H6B—C6—H6C	109.5
O1—Cu1—O5	86.16 (11)	N3—C7—N4	109.4 (3)
N3—Cu1—O5	100.35 (12)	N3—C7—C10	127.0 (4)
O3—Cu1—O5	95.07 (11)	N4—C7—C10	123.6 (4)
N1—Cu1—O5	104.34 (11)	C9—C8—N4	105.6 (3)
C14—O1—Cu1	114.9 (3)	C9—C8—C12	131.6 (4)
C15—O3—Cu1	113.8 (2)	N4—C8—C12	122.9 (4)
Cu1—O5—H51	140.0	C8—C9—N3	108.5 (3)
Cu1—O5—H52	106.7	C8—C9—C13	130.1 (4)
H51—O5—H52	107.4	N3—C9—C13	121.4 (3)
C1—N1—C3	106.3 (3)	C11—C10—C7	117.6 (4)
C1—N1—Cu1	132.3 (3)	C11—C10—H10A	107.9
C3—N1—Cu1	121.2 (2)	C7—C10—H10A	107.9
C1—N2—C2	108.6 (3)	C11—C10—H10B	107.9
C1—N2—H2	125.5	C7—C10—H10B	107.9
C2—N2—H2	125.8	H10A—C10—H10B	107.2
C7—N3—C9	107.0 (3)	C10—C11—H11A	109.5
C7—N3—Cu1	131.0 (3)	C10—C11—H11B	109.5
C9—N3—Cu1	121.9 (3)	H11A—C11—H11B	109.5
C7—N4—C8	109.4 (3)	C10—C11—H11C	109.5
C7—N4—H4	125.3	H11A—C11—H11C	109.5
C8—N4—H4	125.2	H11B—C11—H11C	109.5
N1—C1—N2	110.2 (3)	C8—C12—H12A	109.5
N1—C1—C4	127.8 (3)	C8—C12—H12B	109.5
N2—C1—C4	121.9 (3)	H12A—C12—H12B	109.5
C3—C2—N2	105.9 (3)	C8—C12—H12C	109.5
C3—C2—C6	131.7 (4)	H12A—C12—H12C	109.5
N2—C2—C6	122.3 (3)	H12B—C12—H12C	109.5
C2—C3—N1	108.9 (3)	C9—C13—C3	113.3 (3)
C2—C3—C13	130.3 (4)	C9—C13—H13A	108.9
N1—C3—C13	120.8 (3)	C3—C13—H13A	108.9
C5—C4—C1	113.4 (3)	C9—C13—H13B	108.9
C5—C4—H4A	108.9	C3—C13—H13B	108.9
C1—C4—H4A	108.9	H13A—C13—H13B	107.7
C5—C4—H4B	108.9	O2—C14—O1	124.7 (4)
C1—C4—H4B	108.9	O2—C14—C15	121.0 (4)
H4A—C4—H4B	107.7	O1—C14—C15	114.2 (4)
C4—C5—H5A	109.5	O4—C15—O3	125.3 (4)
C4—C5—H5B	109.5	O4—C15—C14	120.6 (4)
H5A—C5—H5B	109.5	O3—C15—C14	114.2 (4)
C4—C5—H5C	109.5	H6F—O6—H6G	107.5
H5A—C5—H5C	109.5	H7C—O7—H7D	108.5
H5B—C5—H5C	109.5		

### Hydrogen-bond geometry (Å, °)

**Table:**

*D*—H···*A*	*D*—H	H···*A*	*D*···*A*	*D*—H···*A*
O5—H51···O4^i^	0.85	2.58	3.177 (4)	128
O5—H52···O4^ii^	0.85	1.90	2.748 (4)	174
N2—H2···O2^iii^	0.86	2.09	2.943 (4)	171
N4—H4···O7	0.86	2.03	2.847 (4)	158
O6—H6F···O5^iv^	0.85	2.50	3.259 (4)	149
O6—H6G···O4^v^	0.85	2.30	3.057 (5)	148
O7—H7C···O3^vi^	0.85	2.03	2.882 (4)	178
O7—H7D···O6	0.85	1.97	2.818 (4)	177

Symmetry codes: (i) *x*, *y*, *z*+1; (ii) −*x*+2, −*y*+1, −*z*+1; (iii) −*x*+1, −*y*+1, −*z*+1; (iv) −*x*+2, *y*−1/2, −*z*+3/2; (v) −*x*+2, *y*−1/2, −*z*+1/2; (vi) *x*, −*y*+1/2, *z*+1/2.
